# Culture goes East: Mapping the shifting geographies of urban cultural capital through major cultural buildings

**DOI:** 10.1177/00420980241289846

**Published:** 2024-10-29

**Authors:** David Gogishvili, Martin Müller

**Affiliations:** University of Lausanne, Switzerland; University of Lausanne, Switzerland

**Keywords:** architecture, built environment, cultural buildings, cultural capital, cultural flagships, culture, Global East, globalisation, major global cities, politics, 建筑, 建成环境, 文化建筑, 文化资本, 文化旗舰, 文化, 全球东方, 全球化, 全球主要城市, 政治

## Abstract

Culture has become a major component of policies to put cities on the global map. This article traces the shifting geographies of urban cultural capital using the lens of major cultural buildings, such as the Guggenheim Bilbao Museum and the Louvre Abu Dhabi, which cities often mobilise to compete for attention, reputation, tourists and investment. Based on a custom-built database containing 438 major cultural buildings opened worldwide between 1990 and 2019, this article finds a strong growth in the number and total cost of these buildings throughout the three decades, far exceeding global GDP growth. What is more, there is a geographical shift from the established cities of high culture in North America and Western Europe towards Asia, with a particular concentration in China and the Gulf region. The growth of investment in culture and its fast-changing urban geographies urge a more profound integration of culture in urban studies and a deeper consideration of the role of cultural capital in making global cities.

## 摘要

文化已成为让城市在全球版图上获得一席之地政策的主要组成部分。本文从毕尔巴鄂古根海姆博物馆和阿布扎比卢浮宫等主要文化建筑的视角，追溯了城市文化资本的地理变化。这些城市经常动员起来争夺注意力、声誉、游客和投资。根据包含 1990 年至 2019 年间在全球范围内开放的 438 座主要文化建筑的定制数据库，本文发现这些建筑的数量和总成本在过去三十年中强劲增长，远远超过了全球 GDP 的增长。此外，地理分布出现了从北美和西欧成熟的高雅文化城市向亚洲的转移，尤其集中在中国和海湾地区。文化投资的增长及其快速变化的城市地理促进了城市研究中更深入的文化融合，并促使人们更深入地思考文化资本在打造全球城市中的作用。

## Introduction

A large, dark slab, rising like a giant TV screen some 100 m into the air, is one of the latest additions to Hong Kong’s skyline in what is known as the West Kowloon Cultural District. The building, designed by Swiss star architects Herzog & de Meuron, is home to the M+ Museum, a visual arts museum that opened in November 2021 and was immediately included, the following year, in the top 20 of the *Art Newspaper*’s most popular museums, with over 2 million visitors in 2022 (Cheshire and da Silva, 2023). It drew attention for its ambitions to position Hong Kong – one of the top world cities in the global economy (Taylor et al., 2010) – as a cultural and cultured city. The chair of the West Kowloon Cultural District credited the M+ with ‘transforming Hong Kong from a one-time “cultural desert” into an international art hub’ (Tsang, 2021). In a letter to the editor of the *South China Morning Post*, one reader commented that ‘to be a truly great city, Hong Kong needs to go beyond being a business powerhouse or tourist hub and invest in culture’ (Lo, 2021), with the term ‘invest’ indicating how culture has become a commodity.

Large museums, concert halls, theatres and so on represent one materialisation of how culture has become an asset for cities (Evans, 2003; Florida, 2004; Scott, 2008). These major cultural buildings rely on a combination of large size, high cost, iconic architecture and cutting-edge cultural content to differentiate cities in the global interurban competition and allow the accumulation of urban cultural capital. The Guggenheim Museum in Bilbao, opened in 1997, has become a model for turning around urban futures, and major cultural buildings have grown into increasingly coveted urban interventions (Comunian and Mould, 2014; De Frantz, 2005; Patterson, 2022). These buildings, often designed by world-famous starchitects, are meant to advertise the city and the institution (Lindsay, 2016) and stand out in the global media (Alaily-Mattar et al., 2019). Whether it is Louvre Abu Dhabi, Elbphilharmonie (Hamburg), M+ Museum (Hong Kong) or Getty Center (Los Angeles), urban policy-makers globally have sought to emulate Bilbao’s transformation by launching major cultural buildings (Keating and Frantz, 2004; Patterson, 2022).

This article uses the rise of major cultural buildings to understand the formation of urban cultural capital in cities around the world since 1990. It seeks to analyse how iconic major cultural buildings have grown as a phenomenon, how their global geographies have evolved and what cities have emerged as new global cultural cities. Beyond illustrating the fast-changing geographies of global cultural circuits, our findings call for an expansion of the concept of global cities to include culture, and for a notion of global urbanism that recognises the dynamics of non-Western cities in areas such as high culture, long considered the prerogative of the West. For that purpose, this article conducts a diachronic mapping of the opening of new major cultural buildings globally between 1990 and 2019, based on a custom-built database with 438 major cultural buildings. Their total capital cost in 2019 was close to US$84 billion, underscoring the economic weight of this phenomenon and the importance of analysing it.

## Literature review

The past few decades have witnessed the strong growth of the cultural symbolic economy in cities around the world (Scott, 2008). This economy relies on the creation of symbolic value such as meanings and images (Bourdieu, 1971), and affective, atmospheric experiences such as pleasure, joy and fascination (Edensor, 2012). The symbolic and affective power of the cultural sector has become important in inter-city competition as a means of attracting and sustaining global human and economic flows, as cities try to compensate for decaying Fordist production systems while some newly emerged ones are making claim through the same process (Kong et al., 2015; Richards, 2014).

**Figure 1.**
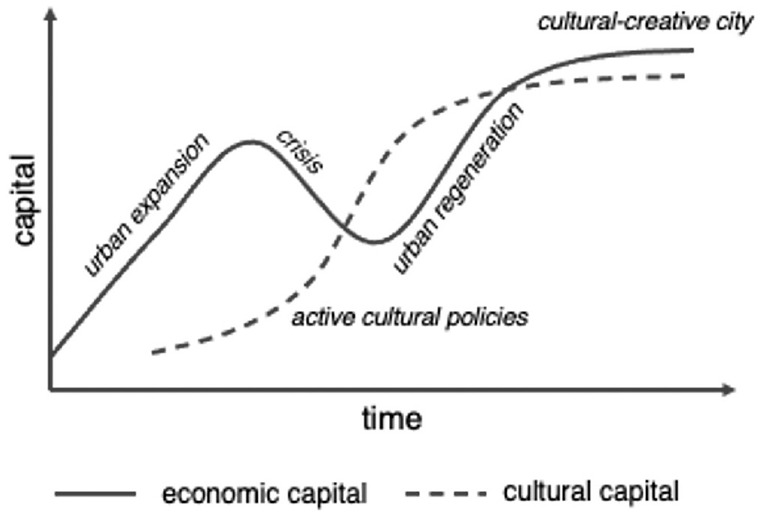
The economic capital/cultural capital cycle of urban development. *Source*: Authors’ own design.

As competition for global economic flows has intensified, cities have invested in the development of new cultural offerings to accrue (urban) cultural capital. Urban cultural capital is here understood as the possession of a recognised offer in the creative, cultural and arts sector that creates distinction from other cities, reformulating Bourdieu’s (1984) famous definition for the purpose of cities (see Savage et al., 2018). As with Bourdieu, urban cultural capital can be converted, to some degree, into economic capital and vice versa (Bridge, 2006b; Johnson, 2006). This is illustrated in stylised form in Figure 1. With the growth of cities, we see the accumulation of economic capital (firms, jobs, tax revenue etc.). This accumulation leads, with a slight delay, to an the increase in cultural capital, as wealthier cities and patrons sponsor museums, galleries and cultural events, converting economic capital into cultural capital. As economic fortunes reverse, for example when deindustrialisation or an economic crisis sets in, cities can deploy active cultural policy, investing in the arts and seeking to convert cultural capital into economic capital. This culture-led urban regeneration has been widely studied (e.g. Evans, 2003, 2005; Grodach, 2008; Lin and Hsing, 2009) and has been at the heart of cases like the Guggenheim Museum in Bilbao (Plaza, 2006). When the promotion of culture makes a city more attractive for investors, tourists and residents, this can then result in an urban area with high stocks of both economic and cultural capital, with a significant role of the cultural and creative sector in the urban economy.

Cultural flagship buildings correspond to ‘objectified’ cultural capital in Bourdieu’s (1984: 17) division of forms of cultural capital, referring to cultural goods and material objects. As concrete-and-glass manifestations, flagship buildings articulate a particular aesthetic and taste, often through spectacular architecture (Plaza et al., 2022; Sklair, 2005) and claim to global distinction, through the ambition to present world-class content in shows and exhibitions. Among a range of options presenting themselves to cities wanting to accrue cultural capital, flagship buildings distinguish themselves by their cost, structural design features and grandeur (Patterson, 2020; Sklair, 2006). Cities compete to contract elite architecture companies for their new or rebranded cultural institutions, expecting to garner sufficient cultural capital to put them on the map of global recognition (Patterson, 2012; Sklair, 2005). Moreover, these buildings are utilised for fashion shoots, movie backgrounds, commercials, music videos, social media and other things, underscoring their role as foils to the wider symbolic economy (Knox, 2011). In this vein, building iconic cultural buildings has become crucial as cities compete to be recognised as a global city (Doel and Hubbard, 2002), with more and more officials believing that economic strength is insufficient for becoming recognised as a leading global city and turning towards culture (and often sports) as a means of distinguishing themselves from the competition (Kong, 2009; Oakes and Wang, 2016; Yeoh, 2005).

Urban studies research, however, has a threefold blind spot when it comes to understanding the role of major cultural buildings in the global competition for cultural capital. First, the acquisition of cultural capital and its links with gentrification, regeneration and urban transformation have mostly been studied at the neighbourhood and city level. By concentrating on these micro-level processes, urban studies has paid less attention to macro-level implications of major cultural buildings in the global circulation of cultural capital. Zukin’s (1995) work on loft living and gentrification in New York City has illuminated how cultural amenities can contribute to neighbourhood change and the displacement of lower-income residents. Bridge (2006a, 2006b), also focusing on the neighbourhood scale, has argued that cultural capital plays a significant role in neighbourhood change, but its impact is not always positive. With the case of Bristol, he demonstrated that the different forms of cultural capital can be conflicting, shaping gentrifiers’ housing choices and leading to diverse neighbourhood trajectories (Bridge 2006a). While Fainstein (2010) has emphasised the importance of culture in urban development strategies, her focus has primarily been on the role of cultural policies and planning, rather than on the specific impact of individual cultural buildings on global city competition. However, this scholarship primarily relies on Western urban experience and theoretical models as reference points for understanding culture, potentially overlooking practices and experiences beyond this geography (Torre, 2024).

The second blind spot can be found in the literature on cities in global networks. While this approach would be able to conceptualise the global reach of cultural capital and its value in interurban competition, it has tended to concentrate on economic capital and power. Sassen (2001) sees global cities as ‘production centres for the inputs that constitute the capability for global control’ (Derudder et al., 2011: 129). She suggests analysing core dynamics between the network of cities rather than the unit of the city as a container. The Globalization and World Cities Research Network’s roster of world cities focuses on advanced producer service firms – accounting, advertising, banking and law (Beaverstock et al., 1999). This approach originates in Sassen’s argument that these firms are the key today to world city formation in North America, Western Europe and regions of Pacific Asia (Derudder and Taylor, 2018). The role of culture in the formation of global cities, however, has remained rather overlooked, with systematic research limited to a few instances (Caset and Derudder, 2017; Skórska and Kloosterman, 2012; Yin and Derudder, 2021) and lacking a diachronic perspective.

The third and final blind spot relates to the spatial distribution of research on culture in cities. Culture, and the acquisition of cultural capital, has so far been mainly considered as a prerogative of Western cities. Studies focusing on Bilbao, London, New York, Paris and Vienna dominate research on the role of culture in cities. This is despite the fact that many cities in Asia ‘striving for global city status have recognized what investing in cultural infrastructure’ brings to their ambitions (Kong et al., 2015: 7). Moreover, often case studies are confined to certain countries or regions of the world, lacking a global, longitudinal and systematic view. Thus, there is limited evidence on how major cultural projects contribute to a range of development or regeneration initiatives (Evans, 2005). This blind spot prevents us from rethinking existing theoretical and empirical knowledge, as most research comes from Western cities. One exception to this trend is the work focusing on Chinese cities that examines the spread of the grand theatres (Xue, 2019a). Other works that focus on cities beyond the West usually discuss only a few cases from China (Beijing, Hong Kong and Shanghai), Seoul, Singapore or Taipei (Chang, 2000; Goudsmit et al., 2024; Kong, 2009; Lee, 2015; Lee and Hwang, 2012).

We lack therefore a comprehensive view of cultural flagships, their global reach and their significance in shaping the formation of cultural capital in and between cities at the global scale. To address these blind spots in our knowledge on the global production of cultural capital, longitudinal research at the global scale is needed to show historical developments and go beyond the predominant focus on case studies. The research in the rest of this article analyses the global urban geographies of major cultural buildings and their change over time. We call for an extension of the concept of global cities to encompass culture and a notion of global urbanism that recognises the dynamics of non-Western cities in sectors such as high culture, long considered a privilege of the West.

## Research design

Our empirical goal for the research design was sought to trace the shifting geographies of major cultural buildings – libraries, multifunctional arts venues, museums and performance venues – over time as one indicator of the global creation of cultural capital in cities. Thus, these buildings and where they are being realised show how the concept of global cities and the forms of capital attached to them are evolving. We aimed to detect patterns and trends across the location, size and cost of these institutions. To delimit our sample, we set temporal and institutional parameters. While a new period of cultural capitalism in cities took off in the 1980s (Harvey, 1989; Scott, 2008), we set the year 1990 as the starting point for our data collection as it marked the beginning of a period of intense globalisation: the Cold War had ended and the countries of the Global South and East had started opening up to the world, while the spread of the internet bound the world ever more tightly together. We ended our data collection in 2019 to avoid the COVID-19 pandemic bias.

We defined a ‘new major cultural building’ as being a new or existing cultural institution with a building inaugurated between 1990 and 2019. To delimit what makes a building a ‘major’ building, we additionally required either a minimum floor space of 20,000 m^2^*or* a realisation cost of at least US$100 million (in 2019 value) *or* a minimum capacity of 1500 persons (for performing venues) for inclusion in the sample. The cost was set to 2019 US$ to adjust for inflation and currency differences. These thresholds orientate themselves at the size of the Guggenheim Bilbao (24,000 m^2^ floor space) and the cost-based definition of major projects (>US$100 million) of Flyvbjerg (2011). All institutions globally that were built within this period and meet one of the criteria are included in our dataset, resulting in a sample of 438 cases in 58 countries with a total cost of US$84.2 billion (in 2019 value). The dataset, with sources for every data point, is available online on Harvard Dataverse free data repository (Gogishvili and Müller, 2024).

In this dataset, we collected information on the name of the institution, city, country, continent, architect, year inaugurated, type (library, multifunctional venue, museum, performance venue), construction cost, floor area and maximum capacity. The data in the dataset were primarily obtained from the websites of cultural institutions and architects. If information was missing, we referred to government websites and reports generated by governments, consultancies and non-profits. This was supplemented by architecture and culture magazines, and academic sources. Similar sources are used by other studies that focus on iconic buildings or starchitecture (Patterson, 2022; Ponzini and Manfredini, 2017; Sklair, 2017).

Due to the iconic status of major cultural buildings and global interest in them, information included in the dataset is widely available, often in English, the primary language used for the data collection. The information structure for all variables was relatively simple. Thus, artificial intelligence-assisted translators were efficiently used for data collection for other languages. Chinese cases presented an exception in terms of data availability, since information was sometimes not available in any language, including Chinese (see the annex of Xue, 2019b). Sixty-eight missing values (56 for cost and 12 for floor space) for 64 institutions (70% from Asia and the majority from China) were imputed for calculating aggregates by assigning the average realisation cost of the major cultural building in the country. Missing values were 12.7% for total realisation cost and 2.7% for total floor space. Out of a total realisation cost of almost US$84.2 billion, the total cost of the imputed cases is US$6.6 billion. There were 13 cases for which missing values could not be imputed, as there was no other major cultural building of that type for the country.

The main limitation of this dataset is in how it allows and does not allow us to understand a cultural building. In its focus on cost and size, it identifies big, expensive buildings with a cultural vocation. It therefore shows the ambition of cities to invest in and promote culture and the desire to accrue cultural capital. Our data are unable to capture more qualitative elements that make a cultural building confer distinction on a city and radiate beyond a city: iconic architecture, for example, or cultural recognition. It is quite possible that many of the buildings in our database do not have the iconic architecture nor the outstanding programming to have global appeal. Thus, we have decided to call unit of analysis ‘major cultural buildings’ rather than ‘cultural flagships’ or cultural ‘landmark buildings’, other terms common in the literature that reference a building’s global recognition or iconic status (e.g. De Frantz, 2005; Grodach, 2008; Patterson, 2020; Plaza et al., 2022). In a parallel study, we are currently investigating questions of the architecture, ownership and visitor appeal of the programming and political dynamics of cultural flagships, to arrive at a more complex picture.

## Analysis

### Global level

**Figure 2.**
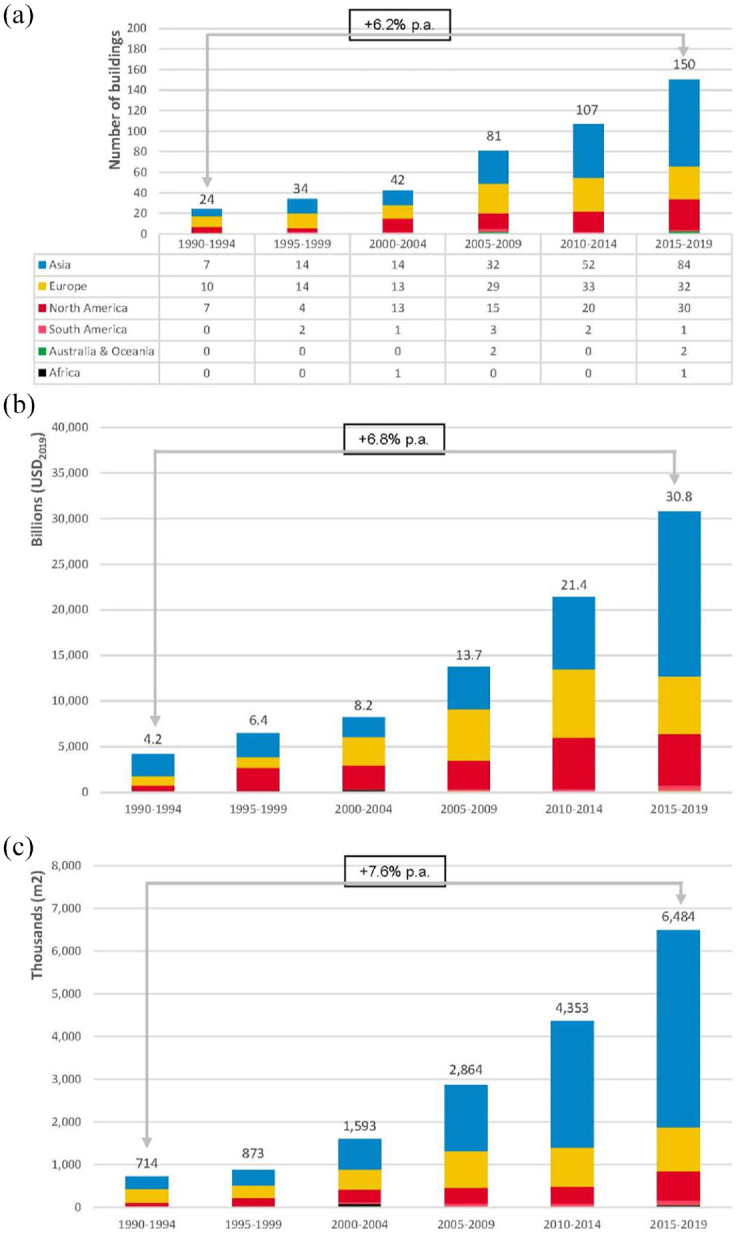
Number of major cultural buildings inaugurated since 1990: (a) by five-year period and continent, (b) by total cost and continent and (c) by total size in square metres and continent.

Our dataset demonstrates the increasing popularity of major cultural buildings globally. As Figure 2 shows, after the 1990–1994 period we have witnessed a growth in the number of new major cultural buildings with each five-year period. While Europe initially led, Asia subsequently surpassed it. This change can be credited to the increased investments in the cultural infrastructure in China (Howarth, 2015; Xue, 2019a), the Gulf (Exell and Wakefield, 2016; Ponzini, 2020) and the Global East (Koch, 2018).

The global financial crisis slowed down the inauguration of major cultural buildings, as is visible in the period from 2010 to 2014, but growth continued and later accelerated. Over 32% of the buildings in the dataset were inaugurated only during the latest five-year period. This can be again attributed mostly to Asia, where more than half of these buildings were built. However, compared to previous periods, the role of North America has risen significantly. Thus, our sample demonstrates how major cultural buildings first became popular in Europe, likely as part of the increasing role of culture in urban revitalisation (De Frantz, 2005; Evans, 2009; González, 2011), and then there was a massive geographical shift towards Asia (Kong et al., 2015; Wang et al., 2015).

Figure 2b shows a general upward trend in the total cost of major cultural buildings. The compound annual growth rate of this variable is 6.8%, which substantially exceeds the average global growth of GDP (2.9%) in the same period. The most expensive institution in the sample is the Getty Center (inaugurated in 1997) in Los Angeles, at a cost of almost US$2 billion (2019 value). The centre is extensive, covering almost 90,000 m^2^, and combines different cultural offerings. The Millennium Dome in London (2000) built at a cost of US$1.63 billion (2019 value) is the second and is followed by the Louvre Abu Dhabi (2017) with a cost of US$1.31 billion (2019 value). The fourth and the fifth are the Fondation Louis Vuitton (2014) in Paris and the Stavros Niarchos Foundation Cultural Center (2016) in Athens. They cost US$1.25 and US$1.1 billion (2019 values) respectively.

The 438 major cultural buildings that have been built over the last three decades have almost 17 km^2^ of floor space altogether. In terms of total floor space, the dominance of Asia is even more significant (Figure 2c). More than 60% of new buildings’ total floor space was created in Asia, not only because of the sheer number of institutions created but also because of their size. The average floor area is also the highest in Asia at almost 48,000 m^2^. In the period of 2015–2019, the share of Asia reached 71%. Considering the ongoing construction of new cultural institutions there, this share will increase further (AEA Consulting, 2021). Out of the 20 largest major cultural buildings, the first 10 are in Asia, mostly in China.

### Country level

**Figure 3.**
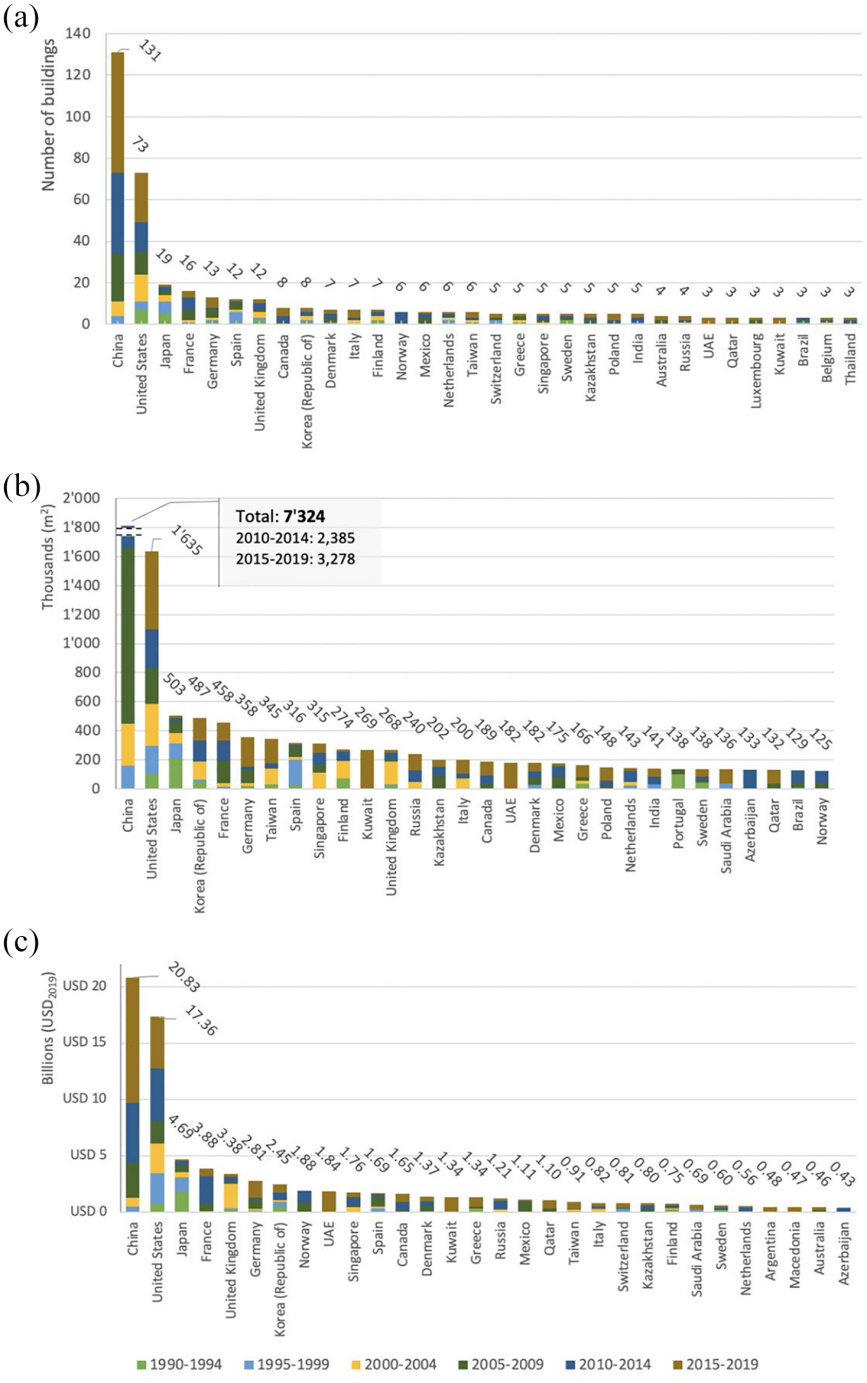
Distribution of major cultural buildings by (a) country and five-year periods (with at least three new buildings), (b) total cost of all institutions by country and (c) total size of all institutions by country.

Disaggregating data by country reveals a more nuanced picture (Figure 3a). Despite Asia’s dominance, due to the salient role of China, the top 10 countries with major cultural buildings are primarily in the Global North. If we look at the countries that have three or more new major cultural buildings, the picture becomes more global.

The strong position of the Global North here is largely an outcome of higher investments before 2005, while after this a more varied set of countries has emerged. For example, 10 out of 14 major cultural buildings built on the Arabian Peninsula have been inaugurated since 2015. This is an illustration of the policy change in countries like Kuwait, Qatar or the UAE, aiming to diversify their economy (Exell and Wakefield, 2016), which is still largely based on revenues from oil and gas exports, as well as their attempt to stake global claims to cultural influence (Giusti and Lamonica, 2023). Hydrocarbon-rich Azerbaijan and Kazakhstan also built large buildings at relatively low cost, since they have started to invest in state-initiated urban megaprojects to put themselves ‘on the map’ (Koch, 2018).

China and the USA – the two largest economies in the world – dwarf all other countries, also in terms of the total cost and total size of their major cultural buildings (see Figure 3). Taken together, they inaugurated 47% (30% and 17%) of major cultural institutions during the last three decades and more than half of them in the latest period. The dominance of Asian countries here can be partly attributed to the large scale of the Grand Theatres built in China, as well as the ‘museum boom’ criticised as an attempt to ‘buy culture’ (Howarth, 2015), which has seen a particular rise in the last two decades. The supremacy of China is unparalleled and is likely to continue further into the next decade, as many new projects are underway in cities like Jinan, Shanghai and Shenzhen (AEA Consulting, 2021). The latter had at least 10 ongoing projects of almost US$3 billion in 2021 (AEA Consulting, 2021: 6). State ownership of land in many Asian countries is likely to be a main driving factor in creating such large structures. In addition, the political regimes of many emerging states seek to make a statement through the iconicity and grandeur of large public buildings.

There is a shift in the ranking if we look at the data by size. Many countries from the Global East or South move up the ladder (Figure 3b). Thus, despite the sometimes lower number or cost of major cultural buildings in these countries, the buildings are often large. For example, the two major cultural buildings with around 80,000 m^2^ total floor space, the Museum of Contemporary Art & Planning Exhibition Shenzhen and Paris Philharmonic, which were inaugurated in 2015 and 2016 respectively, incurred radically different costs. The concert hall in Paris cost around US$450 million, while the museum in Shenzhen cost three times less (AEA Consulting, 2017). The average size of projects in the USA and France is at least two to three times lower than that of projects in Qatar, South Korea and Taiwan. The dominance of large-scale buildings such as the National Museum of Qatar in Doha, the Asia Culture Center in Gwangju or the National Kaohsiung Center for Arts in Kaohsiung City underscores the strive towards grandeur and iconicity.

Although China is currently leading, the margin is significantly smaller when countries are ranked based on the total cost. When countries with at least three projects are compared based on cost per square metre created, China ranks towards the bottom of the list, as it spent approximately US$2500 (2019 value) (Table 1). The USA, which ranked second by the total number, size and cost, also ranks third in the top 10 countries by the cost per m^2^. Norway dominates this list, where the construction of one flagship cost over US$15,000 (2019 value). Out of the top 10 where m^2^ of floor space costs most, only two are from outside the Global North: Qatar and the UAE. These two countries, with Kuwait, have paid the highest amount per building.

**Table 1.** Countries ranked based on the cost per m^2^ and the average cost of building.

Countries ranked by average cost per square metre in US$ (2019 values)		Countries ranked by average cost per building in million US$ (2019 values)	
Norway	15,078	UAE	613
United Kingdom	12,605	Kuwait	448
United States	10,615	Qatar	365
Switzerland	10,613	Singapore	351
Australia	10,370	Norway	314
UAE	10,106	Korea (Republic of)	306
Japan	9320	Russia	303
Canada	8746	United Kingdom	282
France	8459	Greece	268
Qatar	8290	Japan	247
Greece	8030	France	242
Germany	7836	United States	238
Denmark	7527	Germany	216
Mexico	6338	Canada	206
Luxembourg	5681	Denmark	195
Singapore	5585	Mexico	185
Spain	5337	Switzerland	161
Russia	5045	Kazakhstan	159
Korea (Republic of)	5029	China	159
Kuwait	4998	Taiwan	151
Sweden	4326	Spain	141
Italy	4113	Luxembourg	123
Kazakhstan	3952	Sweden	119
Netherlands	3916	Italy	118
China	2846	Australia	115
Finland	2730	Brazil	110
Poland	2681	Finland	107
Taiwan	2632	Netherlands	93
Brazil	2552	Poland	79
Belgium	2505	Belgium	43
India	1197	India	34
Thailand	881	Thailand	26

The geography of the most expensive major cultural buildings realised between 1990 and 2019 is rather different. Many of the most costly projects are in the Global North – the Getty Center (Los Angeles), the O2 Arena (London), Fondation Louis Vuitton (Paris), the Stavros Niarchos Foundation Cultural Center (Athens) and Elbphilharmonie (Hamburg). In Asia, except for the Gulf, they are building significantly larger venues at a lower price. The reasons for this need to be explored in further research.

### City level

**Figure 4.**
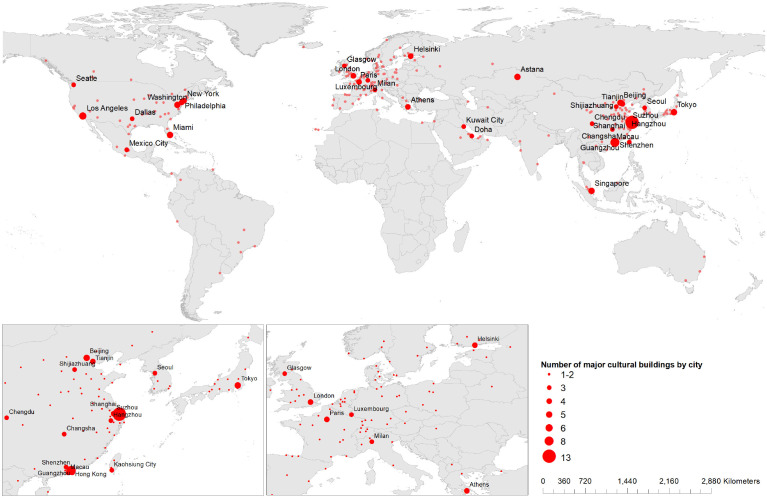
Distribution of new major cultural buildings by city from 1990 to 2019. *Note*: Only the cities with a minimum of three new buildings have a label.

The map in Figure 4 conveys the geographical distribution of all the cities that inaugurated major cultural buildings from 1990 to 2019. There is a strong concentration in a small number of cities. Cities with at least three new buildings (33 cities) are home to 139 institutions. The list is led by Shanghai (13 buildings), Shenzhen (eight) and Los Angeles (six). None of them is a capital city. Shanghai has made the most remarkable progress, illustrated by the amount of new cultural infrastructure and by its growth. The next are major cities with five new cultural buildings each – Astana, Beijing, Miami, Singapore, Tokyo and Washington DC. In contrast, in Miami, private actors play a key role as initiators, supporters or owners of these institutions. In others, it is usually governments that are the driving force behind such initiatives. Major global cities like Los Angeles, Paris and London, known for their cultural infrastructure, have fewer new institutions but they spend the most overall.

Unsurprisingly, the majority of these cities are on the roster of the Alpha World Cities by the Globalization and World Cities (GaWC) research network (GaWC, 2020). Astana and Miami are two exceptions, as they score ‘Sufficiency’ and ‘Beta+’. The presence of Astana can be explained by Kazakhstan’s former president’s affection for spectacular urban development (Koch, 2018), while Miami has grown into the centre of the entertainment and arts industries in the USA. Notably, this dataset is not entirely aligned with ‘the uneven geographies of economic power focused in the Global North’ (Derudder and Taylor, 2018: 1030), led by London and New York, which often top various city rosters. This duo occupies unusual positions in our database by not being at the top. This may be due to their longstanding and well-established cultural infrastructure.

There are only three Western cities, all from the USA, that have at least five new major cultural buildings: Los Angeles, Miami and Washington, DC. They are home to major art fairs, Art Basel Miami and Los Angeles Art Show, and both old and new major cultural institutions such as the Arsht Center for the Performing Arts (Miami), the Getty Centre (Los Angeles), the Museum of the Bible (Washington), Perez Art Museum (Miami), the Smithsonian Museum of African American History & Culture (Washington) and the Walt Disney Concert Hall (Los Angeles).

Doha and Kuwait City, with three major cultural buildings each, are the only cities from the Gulf in this list. This is different compared to a roster of world cities by advanced producer services, where we also see Abu Dhabi, Dubai and Riyadh (Beaverstock et al., 1999; GaWC, 2020). This list will include more of the region’s cities soon, as ongoing major cultural projects such as Abu Dhabi’s Saadiyat Island Cultural District are completed (Architectural Record, 2022).

Hong Kong, Shanghai and Singapore – all among the cities with the highest number of new buildings – have all been investing in buildings to accumulate cultural capital ‘as part of the strategy to help their cities gain global city status’ (Kong, 2009: 1). Shanghai has been one of the first in Asia to apply creative economy strategies to recover from the Asian financial crisis (Kong et al., 2015). The government has been aiming for a cultural revival and has ‘a desire to gain primary position in the national imaginary’ and to achieve ‘global city’ status (Kong, 2007: 394). The most visible manifestation of this policy is Shanghai Grand Theatre, designed by Arte Charpentier Architects and inaugurated in 1998. It is complemented by the Oriental Art Centre, by Paul Andreu, and the Shanghai Science and Technology Museum, one of China’s most visited cultural venues.

## Conclusion

In this article, we have mapped the increasing global geographical coverage of new major cultural buildings as an important component in the globalisation of cities over the latest three decades. We have shown that major cultural buildings have experienced strong growth since the 1990s. The investment volume in major cultural buildings has increased fivefold from the 1990s to the 2010s, rising from US$10.6 billion to close to US$52.2 billion (2019 values). This increase far outstrips the growth of global GDP in the same period. Our systematic database shows the superlinear growth of the cultural economy in the case of major cultural buildings. It therefore lends support to Engels’ Law for the cultural sector, which holds that as disposable income expands, consumption of symbolic goods rises at a disproportionately higher rate (Scott, 2008: 84–85).

The exorbitant sums sunk into major cultural buildings underscore the competitive aspect of obtaining cultural distinction. The allocation of economic capital above and beyond what appears as rational from a purely economic standpoint may constitute a form of overconsumption of prestige commodities to obtain social status, observed by Veblen (1899; see Bridge, 2006b). In this explanation, cities splurge on cultural buildings because extravaganza is more effective in obtaining global distinction than incremental, modest investments. Given the high capital cost of these buildings, such a strategy is only available to large, wealthier cities. This implies a geography of urban cultural capital marked by high concentration in few metropolitan centres. In this sense, major cultural buildings constitute a marker of (world-)class distinction much akin to Bourdieu’s idea of cultural capital.

Furthermore, our results demonstrate that the pursuit of urban cultural capital has shifted to the East: while Europe was dominant in the opening of major cultural buildings until the 2000s, Asia has since far overtaken all other regions, with a particular concentration in China and, more recently, the Arabian Peninsula. At a more conceptual level, this geographical shift in the construction of major cultural buildings can be understood as an attempt by cities in emerging economies to convert (relatively abundant) economic capital into (relatively scarce) cultural capital. Such conversion is far from straightforward and is constrained by existing cultural hierarchies (Bissenova, 2014; Exell, 2017). Cultural actors from the Global North still hold a large part of the definitional power, as embodied cultural capital of expertise and accumulating urban cultural capital require the reevaluation of what and where counts as culture and cultured.

The strong growth and shifting geographies of major cultural buildings have three profound implications for urban studies. First, culture needs to be taken more seriously and given more weight in global cities research, as it is one of the most strongly growing sectors of urban economies today. The relationship between cultural capital and economic capital is complex, and the possibilities and constraints around converting one into the other suggest examining the two together. Second, the geographical shift towards the East lends further arguments to calls to give more attention to non-Western cities (Ren, 2021; Robinson, 2016; Roy, 2009). Our analysis allows us to see a batch of new global cities of culture emerge, such as Shanghai, Shenzhen, Seoul, Singapore, Astana, Kuwait City and Doha – cities that cut across established divides of Global North and Global South and call attention to the ‘missing East’ in global urban studies (Kong and Qian, 2019; Müller and Trubina, 2020; Wang, 2020).

Third and last, the geographical shift towards the East also spells out the need to interrogate long-held theoretical explanations. Much research in the West links the role of culture in cities to urban regeneration, entrepreneurialism and the global competition for talent, tourism and investment. Yet, these conceptual frames may need revision in cities of the East (Chang, 2000; Kong et al., 2015; Torre, 2024), where the state may play a more significant role, the scale of reference may be more national than global and political considerations of soft power and regime legitimation through cultural capital accumulation may outweigh economic rationales. The quantitative research in this article has prepared the ground for further enquiries to place culture at the centre of global urban studies and give adequate attention to its varied global geographies and theoretical explanations.
